# Objectively-measured physical activity in children is influenced by social indicators rather than biological lifecourse factors: Evidence from a Brazilian cohort

**DOI:** 10.1016/j.ypmed.2016.12.051

**Published:** 2017-04

**Authors:** Alan G Knuth, Inácio Crochemore M Silva, Vincent T van Hees, Kelly Cordeira, Alícia Matijasevich, Aluísio J D Barros, Iná Santos, Ulf Ekelund, Pedro Curi Hallal

**Affiliations:** a Federal University of Rio Grande (FURG), Postgraduate Program in Public Health, Brazil; b Federal University of Pelotas (UFPel), Postgraduate Program in Epidemiology, Brazil; c Department of Sport Medicine, Norwegian School of Sport Sciences, Oslo, Norway; d Medical Research Council (MRC), Epidemiology Unit, Institute of Metabolic Science, University of Cambridge, Cambridge, UK; e Department of Preventive Medicine, University of São Paulo, São Paulo, Brazil

**Keywords:** PA, physical activity, DOHaD, Developmental Origins of Health and Disease, ALSPAC, Avon Longitudinal Study of Parents and Children, Pediatrics, Physical activity assessment, Health determinants

## Abstract

The aim of this study was to examine the longitudinal influences of early life social and biological indicators on objectively measured physical activity. All newborns in 2004 in the city of Pelotas, Southern Brazil were enrolled in a birth cohort study. At the age of 6 years, a follow-up visit included objective assessment of overall physical activity (summarized in milli-g, 1 mg = 0.001 g) by tri-axial wrist worn accelerometry. The associations between early life exposures, such as type of delivery, parity, birth weight, preterm delivery, maternal physical activity, socioeconomic position, and overall physical activity were examined. Valid accelerometry data were obtained from 2604 children (78.2% of the eligible individuals). Girls were less active than boys (β = − 8.65 mg; 95% CI − 10.0; − 7.30). Higher socioeconomic position was related to lower activity levels (β = − 9.69 mg. 95% CI − 12.45; − 6.93) and a similar association was found with maternal schooling. No associations were found with birthweight, type of delivery or preterm delivery. This study provides evidence for the role of some social factors in explaining children's physical activity behaviors, and minimizes the influence of some early life biological factors at determining physical activity levels.

## Highlights

• There was no biological role of early life variables on physical activity behaviors.
• There was a strong evidence on the role of social factors.
• Higher socioeconomic position was associated to lower physical activity levels.

## 1 Introduction

Accurate measurement of physical activity is a challenge in all age groups, but particularly in childhood. Self-report instruments based on the perception that mothers or guardians have about children's physical activity levels (proxy reports) and those based on self-reports from the child are subjective measure. Given the sporadic nature of the movement at this age group (Rowlands, 2007), assessing physical activity is particularly demanding at this age, and proper measurement is a starting point for the epidemiological evidence on physical activity levels of children around the world.

Practical issues, such as the costs associated with the use of criterion standards, i.e. doubly labeled water, which estimates physical activity based on energy expenditure, make it difficult to use such instruments in large scale studies, leading researchers to rely on secondary or subjective measures of physical activity (Sirard and Pate, 2001). From the secondary measures available, accelerometry is the most widely used method as it is possible to use in large scale studies including those with children. Some of the important issues of accelerometry at this age group (Rowlands, 2007) are related to: (a) the epoch (the signal from an accelerometer summarized over a given time interval), that must be short; (b) the duration of measurement, that shall include weekends; (c) the time of use, that shall include night and day avoiding replacements of the device; and (d) the position of use, hip or wrist. In order to determine the overall physical activity and the context in which it takes place, a combination of accelerometry and a subjective instrument is desired.

There are two important gaps in the knowledge about physical activity in children: the limited prospective data available and the lack of in-depth analyses of the influence of some social variables on physical activity behaviors. Studies on physical activity in the context of the Developmental Origins of Health and Disease (DOHaD) hypothesis are even rarer (Ridgway et al., 2011, Hallal et al., 2006). One study combining data from European and Brazilian cohorts concluded that none of the birth variables was associated with physical activity at 11–12 year-olds (Mattocks et al., 2008). Another study showed social and ecological exposures tend to influence physical activity levels among adolescents (Hearst et al., 2012). Among low- and middle-income countries, insights about the social determinants of health are essential, given the socioeconomic inequalities (Paim et al., 2011). Therefore, the Brazilian context or similar places add knowledge in terms of a mix, comprehending social and cultural expression of the behaviors. The Brazilian case is of particular interest, given the country has a longstanding history of high socioeconomic inequalities, especially in terms of income inequality.

The aims of this study were to (a) describe objectively-measured physical activity among children aged 6 years participating in a birth cohort in a middle-income setting; (b) explore associations of some biological and social variables, collected early and contemporary in life, with current physical activity levels.

## 2 Materials and methods

All live births in 2004 in the city of Pelotas (South of Brazil) from mothers residing in the urban area of the municipality were eligible to participate in a cohort study (*n* = 4231 children). Mothers were interviewed within 24 h after delivery and infants were weighed and measured. Participants were seen at the ages of 3 months (*N* = 3985), 1 (*N* = 3907), 2 (*N* = 3869) and 4 years (*N* = 3799) prior to the follow up visit at 6 years.^8^ Data on child sex, birth weight, gestational age, and type of delivery, as well as parity (birth order) and self-reported leisure-time physical activity of the mothers before and during pregnancy were collected in the perinatal study. Mothers were classified as active in pregnancy if reporting any physical activity practice in all three trimesters of gestation. When children were 4 years old, self-reported leisure-time physical activity of mothers and the mothers' perceptions about physical activity of children compared to their peers were assessed. Maternal education was collected through completed years of schooling at the 6 (mean: 6.8) year follow-up, and socioeconomic position at birth was assessed through an asset index, later divided into four groups, from 1 (the wealthiest) to 4 (the poorest).

At the 6 year follow-up (Santos et al., 2014), children and their parents were invited to the research clinic of the Epidemiologic Research Center, Federal University of Pelotas for a comprehensive assessment of health. This fieldwork lasted for 10 months, from October 2010 to August 2011. A detailed description can be found at Santos et al. (2014).

At the research clinic, children and their parents received instructions regarding the use of the accelerometer, which was used from 4 to 7 days according to the following protocol: children who were assessed on Mondays, Tuesdays or Wednesdays were asked to wear the accelerometer until the following Monday. Those who were seen on Thursdays, Fridays or Saturdays used the accelerometer until the following Wednesday. The methodological approach was based on the number of accelerometers, availability of research assistants to collect the monitors and to guarantee at least weekend day of measurement. The monitors were placed on the non-dominant wrist of the children and the following instructions were given: a) use the monitor at all times of the day, even while sleeping and bathing, b) contact the research team if you have any questions, and c) schedule a date and time for the return of the device. A research team member collected the device at the scheduled time at the children's place.

Overall physical activity was measured by the GENEActiv accelerometer model to collect acceleration data (1 *g* = 9.8 m·s^− 2^, here the data are expressed in mg - 1 mg = 0.001 g) in three axes. These devices are waterproof and lightweight (16 grams), and were set-up with a frequency of 85.7 Hz. The commercial GENEActiv software was used to initialize and download data from each accelerometer monitor. The accelerometer data in binary format was analyzed with R-package GGIR (http:/cran.r-project.org) (van Hees et al., 2013), providing the main summary measure with the average magnitude of wrist acceleration over the measurement period (normalizing for missing data, i.e. monitor non-wear). The signal processing scheme (1) verifies the sensor calibration error using local gravity as a reference, (2) detects sustained abnormally high values and non-wear periods, (3) calculates the vector magnitude of body acceleration using the Euclidian Norm minus one (ENMO: $\;\sqrt{x^{2}+y^{2}+z^{2}}-1\;g$) with resulting negative values rounded up to zero, and (4) imputes invalid data segments by the average of similar time points on different days of the measurement (van Hees et al., 2013). Further, the first 10 h and the last 20 h of data in each raw accelerometer file were excluded as these were potential periods when the accelerometers would not be attached to the participants (between initialization and attachment, and between collection of the monitors and download, respectively). Files were considered appropriate for analyses if valid data were present for every 15-minute period in a 24-hour cycle (even when scattered over multiple days) and with calibration error (deviation from 1 g during no movement) lower than 0.02 g (after calibration). For the current analysis, only participants who provided at least two days of measurement were considered.

Statistical analyses were performed using Stata 12.0. Analyses on the influence of early life exposures on physical activity at 6 years were run using one-way analysis of variance in the unadjusted analysis and linear regression in the adjusted analysis. A hierarchical conceptual model was used to include variables in a linear regression model. Initially, birth-related variables were included followed by socioeconomic position, maternal physical activity during pregnancy and finally variables collected when children were aged 4 and 6 years, respectively. A cut-off point of 0.05 was used for statistical significance, but all variables with a *P* value ≤ 0.2 were kept in the model in order to minimize residual confounding. In the analysis between current maternal schooling and physical activity, there was no adjustment for early life socioeconomic position due to the collinearity between the two variables.

Mothers provided their written consent for study participation, this includes the mothers and children information. The study protocol was approved by the Federal University of Pelotas Medical School Ethics Committee.

## 3 Results

Descriptive data are presented in Table 1. In 2004, more boys than girls were born (51.9% vs 48.1%, respectively) and the normal delivery was more prevalent (55.5%) than caesarean. Low birthweight and preterm delivery were 10.0% and 14.5% of the sample, respectively. Mothers became less active during pregnancy, and 63.6% of them perceived their children as active at 4 years old. In the last follow-up visit, around half of the mothers reported complete Elementary school.

Analytical sample was considered according the availability of valid accelerometer data and its characteristics, also presented in Table 1, are similar to the original cohort participants. The research team delivered accelerometers to 3331 cohort members and obtained valid data from 2604 children (78.2%). Reasons for incomplete data included failure to comply with the analytical requirements presented in the methods section and because 514 (15.4%) of the data were obtained with the GENEA accelerometer device. The GENEA accelerometer is a non-commercial model that was used in the beginning of the fieldwork but showed limited applicability at large cohort studies. The GENEA accelerometer was replaced by the current commercial version GENEActiv.

The average number of days of accelerometer wear was 4.7 days (SD = 0.99). In Table 2, accelerometry data obtained using GENEActiv are presented in detail.

**Fig. 1:**
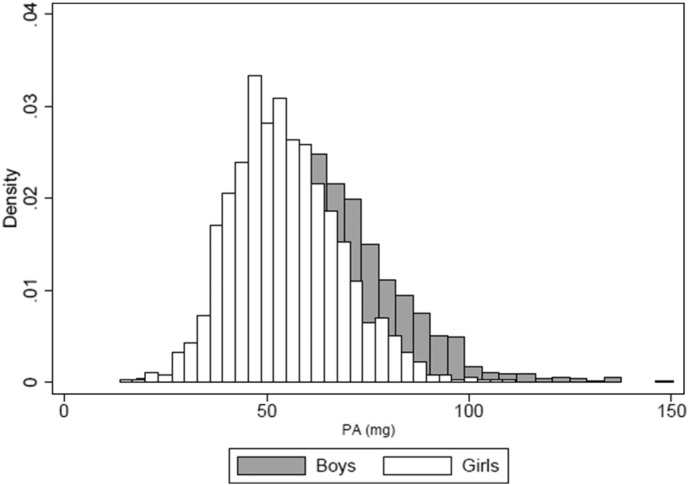
Distribution of overall physical activity (PA) among boys and girls. 2004 Pelotas (Brazil) Birth Cohort Study.

Fig. 1 describes the comparison of the distribution of overall PA in boys and girls. The histograms were positively skewed; however, higher levels of overall PA were identified among boys. Table 3 displays the unadjusted association between early life variables and children's physical activity. Normal delivery, higher parity and poorest socioeconomic groups were positively associated with higher levels of overall physical activity. Children's overall physical activity was higher among those from mother who did not practice physical activity during pregnancy and 4 years after delivery. Maternal schooling was inversely associated with physical activity (Table 3).

In the adjusted analyses, no associations were found between overall children's physical activity and early biological variables and they were not significant to maternal physical activity at pregnancy and at 4 years of children (Table 4). Only three variables remained associated with the child's physical activity: boys were more active than girls and there was a negative and linear association between socioeconomic variables (socioeconomic position and maternal schooling) and overall physical activity.

## 4 Discussion

This study provides evidence for the role of social factors in explaining children's overall physical activity behaviors, and minimizes the influence of early life biological factors at determining physical activity levels in young children. Collecting and describing accelerometry data in over 2500 children from a middle-income setting helps to fill a key literature gap (Hallal et al., 2012). Additionally, the accelerometry data collected for this study will be used as an exposure variable for analyses of the same cohort in future studies. Data are now available with the same accelerometer (GENEActiv) for over 4000 individuals aged 18 years and over 3000 individuals aged 30 years belonging to the 1993 and 1982 Pelotas cohorts (Knuth et al., 2013), respectively, which will allow the investigation of differences in physical activity levels across these three age ranges and its determinants.

A key finding of this study is the clear lack of association between biological variables in early life and children's overall physical activity. The same null finding was observed among Brazilian adolescents based on self-reported data (Hallal et al., 2006) and among British adolescents based on accelerometry data (Mattocks et al., 2008). It should be highlighted, however, that there is a compelling body of evidence supporting the DOHaD hypothesis, which suggests that early life biology influences later health, primarily the development of chronic diseases (Paim et al., 2011). Taken together, our findings suggest that the pathway by which early life factors influence later health is not mediated by reduced physical activity.

A previous meta-analysis using data from 43,482 Nordic individuals showed that those from extreme groups of birthweight had reduced leisure time physical activity levels in adolescence or adulthood (Andersen et al., 2009). The biological plausibility of an association between low birth weight and physical activity could be related to impaired muscle strength and aerobic capacity (Andersen et al., 2009), therefore, suggesting that those who were low birth weight are prone to tiredness. On the other end of the spectrum, children of high birthweight would be more likely to be obese adults, increasing the risk of physical inactivity. These findings were not replicated in this cohort. Finally, a study which used pooled effects from three cohorts from Europe and one from Brazil found no consistent association between birthweight and objectively measured physical activity in 9–15 year-old children (Ridgway et al., 2011).

Birth order is an increasingly studied variable in the DOHaD context. Previous studies have shown that firstborns are more likely to present low birthweight, to catch-up rapidly after birth, and to present increased risk of chronic diseases in adolescence or adulthood (Siervo et al., 2010, Barker et al., 1993). The analyses of both the 1993 Pelotas Birth Cohort and the Avon Longitudinal Study of Parents and Children (ALSPAC) data showed later borns to be more active than firstborns (Hallal et al., 2006, Mattocks et al., 2008). This finding was not replicated in the final model in this study, suggesting that a possible association between birth order and physical activity may not become evident until after adolescence.

In terms of the social determinants of health, Brazil presents a special context for investigation. One fifth of the Brazilians are functionally illiterate, the Gini index for income inequality is 0.55 and marked regional disparities are observed (Paim et al., 2011). This study showed an inverse association between overall physical activity measured through accelerometry and socioeconomic position, similar to the findings by Mattocks and colleagues using the ALSPAC cohort data (Mattocks et al., 2008, Riddoch et al., 2007). Two recent reviews compiled studies on the association between socioeconomic indicators and physical activity. In the articles reviewed by Stalberg and coworkers, a positive association was identified among adolescents (Stalsberg and Pedersen, 2010). It is important, however, to consider that different measures of socioeconomic factors, such as schooling, income, asset indexes, among others, were used (Stalsberg and Pedersen, 2010). Contrary to our study, a 2002 review on risk factors for coronary disease among UK children and young adults (Batty and Leon, 2002) concluded that there was no clear social patterning of physical activity. However, among countries of different income status, a recently published study using data from 122 countries showed that physical activity levels among adults tend to be higher in low-income countries as compared to high-income ones (van Hees et al., 2013). Possibly the effect of social variables on physical activity assumes a distinct interest according to individual possibilities, life choices and the relationship with parents and peers. In the particular case of children as they are not able to completely manage their lives, so the social context and behaviors are defined according to the limits and to their low autonomy to take some actions. The different physical activity domains, social variables, parents´ culture and rearing might act with some dispositive that could change the explanations and the meaning of the children's movements.

Several factors influence a possible association between social indicators and physical activity behaviors. First, there is no universal pattern for this association as it is not merely directional, being mixed with cultural aspects and different contexts. Second, physical activity may be practiced in different domains and with different purposes which impact its correlates. For example, it has been previously shown that the factors associated with leisure-time walking are markedly different from the correlates of walking for commuting purposes (Hallal et al., 2005). In this study, 2/3 of the children walk to school, and these walks last 10 min or more in 64% of the cases. Therefore, children's physical activity comes from other activities, including commuting and household chores, in addition to playing or participating in Physical Education classes (Palma and Assis, 2011). In order to explore the activities in different contexts of one's daily routine, future studies would benefit from complementing accelerometry information with diaries, or other alternatives. One possibility would be using a diary for specific ‘parts’ of the day, for example, school and afterschool time. This would minimize the burden to participants of filling a diary, and would also provide researchers an idea on where the child was during the activities captured by the device.

Positioning the accelerometer in the wrist has several practical advantages as compared to using it in the hip. Most notably, compliance with the protocol (i.e. time of use per day) is increased. During childhood, individuals have the highest levels of physical activity (Mattocks et al., 2008, Corbin et al., 2004). However, not only the behaviors are quite intermittent but also a sporadic nature of the activity patterns have been identified (Rowlands, 2007). Although accelerometry data can more accurately measure these behavioral patterns than questionnaires, data collection has been a challenge. The use of accelerometers on the hip has resulted in the loss of data and accelerometer devices. Nevertheless, by choosing to place the accelerometer on the wrist of children instead of the hip, the handling of the monitors due to use on the hip, such as loss at bedtime, play or change clothes, and in water activities when the device could be hampered may be avoided. In a laboratory study conducted by Phillips et al. the GENEA device presented high agreement via calibration with oxygen consumption and accurately assessed children's physical activity intensity, in the wrist or hip (Philips et al., 2013). Laboratory conditions may not reflect the challenge of physical activity assessment under free-living conditions and the age group was of 8–14 years, different from the present study. Another validation study helps to reinforce the utilization of accelerometer in the wrist in children of 8–10 years (Ekblom et al., 2012).

Some limitations of the study should be discussed. Accelerometer data expressed in mg is not yet easily translated into real life recommendations, an issue that needs to be addressed in the near future. Information on maternal physical activity was collected through the application of a single-item question based on self-report. If misclassification of maternal physical activity is high, dilution of a possible association with children's physical activity will occur. This may explain our lack of association between maternal and child physical activity. Although a simple question on children physical activity at 4 years of age based on maternal perception was used, a previous study showed that this single-item has reasonable agreement with accelerometry data (Bielemann et al., 2011) Lastly, some information on overall physical activity has been lost due to accelerometer device problems. The GENEA device was problematic and impacted negatively the beginning of the study. The lost information was random and was not related to some specific indicator (Table 1), as it affected all cohort members in that moment of the fieldwork.

## 5 Conclusion

By objectively measuring physical activity in a sample of children living in Brazil, this study adds to current knowledge of physical activity levels and its determinants worldwide. This study provides evidence of the absence of biological role of early life variables at explaining physical activity behaviors of children and highlights strong evidence on the role of social factors.

## Conflict of interest

None declared.

## Funding

The 2004 birth cohort study is currently supported by the Wellcome Trust through the programme entitled Major Awards for Latin America on Health Consequences of Population Change (Grant no. 086974/Z/08/Z). The World Health Organization (Grant no. 03014HNI), National Support Program for Centers of Excellence (PRONEX) (Grant no. 04/0882.7), Brazilian National Research Council (CNPq) (Grant nos. 481012-2009-5; 484077-2010-4; 470965-2010-0; and 481141- 2007-3), Brazilian Ministry of Health (Grant no. 25000.105293/2004-83), and Children's Pastorate have supported previous phases of the study.

## Figures and Tables

**Table 1:** Description of social and biological variables among children and their mothers. 2004 Pelotas (Brazil) Birth Cohort Study.

Variables	Original cohort (*N* = 4231) (%)	Analytical sample^a^ (*N* = 2604) (%)
*Variables collected at birth*		
**Sex**		
Boys	2196 (51.9)	1341 (51.5)
Girls	2035 (48.1)	1262 (48.5)
**Type of delivery**		
Normal	2348 (55.5)	1429 (54.9)
Caesarean	1883 (44.5)	1174 (45.1)
**Low birthweight (< 2500 g)**		
No	3804 (90.0)	2379 (91.4)
Yes	424 (10.0)	224 (8.6)
**Preterm delivery**		
No	3603 (85.5)	2263 (87.0)
Yes	612 (14.5)	338 (13.0)
**Parity**		
1	1666 (39.4)	1052 (40.4)
2	1111 (26.2)	672 (25.8)
3	680 (16.1)	417 (16.0)
4 +	773 (18.3)	461 (17.7)
**Socioeconomic position**		
1 (wealthiest)	580 (17.8)	356 (17.2)
2	1128 (34.5)	718 (34.6)
3	1230 (37.7)	805 (38.8)
4 (poorest)	327 (10.0)	195 (9.4)
**Maternal PA before pregnancy**		
Yes	645 (15.2)	398 (15.3)
No	3586 (84.8)	2205 (84.7)
**Maternal PA during pregnancy**		
Yes	185 (4.3)	104 (4.0)
No	4102 (95.7)	2499 (96.0)
*Variables collected at 4* *years of age*		
**Maternal PA**		
Yes	617 (16.3)	411 (16.3)
No	3165 (83.7)	2113 (83.7)
**Maternal perception of child's PA**		
Active	2414 (63.6)	1608 (63.5)
Inactive	1382 (36.4)	925 (36.5)
*Variables collected at 6 years of age*		
**Maternal schooling**		
No schooling	44 (1.2)	26 (1.0)
Elementary school	1762 (48.4)	1252 (48.8)
High school	1327 (36.4)	943 (36.8)
University degree	509 (14.0)	343 (13.4)

PA – Physical activity.

a Participants at 6-year follow-up who provided valid accelerometer data.

**Table 2:** Description of accelerometer (GENEActiv) variables. 2004 Pelotas (Brazil) Birth Cohort Study.

Variables	N
Sample size (valid data)	2604
Missing values	727^a^
Total measurement duration (mean days ± SD)	5.98 (0.99)
Total measurement according protocol^b^ (mean days ± SD)	4.73 (0.99)
Non-wear time (mean hours ± SD)	1.32 (6.70)
Acceleration variability (interquartile interval - mg)	47.6–68.8

SD: standard deviation.

a 514 values from GENEA accelerometer were excluded and 213 children did not comply with the research protocol.

b Total measurement duration considering non-wear time and the deleted initial and final period.

**Table 3:** Predictors of overall physical activity (PA) at 6 years. 2004 Pelotas (Brazil) Birth Cohort Study.

Variable	N	Overall PA (mg ± SD)	P
*Variables collected at birth*			
**Sex**			< 0.001
Boys	1341	63.8 ± 17.4	
Girls	1262	54.7 ± 13.5	
**Type of delivery**			0.01
Normal	1429	60.1 ± 16.4	
Caesarean	1174	58.5 ± 16.1	
**Low birthweight (< 2500** **g)**			0.79
Yes	224	59.7 ± 15.5	
No	2379	59.4 ± 16.3	
**Preterm delivery**			0.36
No	2263	59.3 ± 16.2	
Yes	338	60.1 ± 17.0	
**Parity**			< 0.001
1	1052	58.4 ± 15.4	
2	672	59.2 ± 15.9	
3	417	58.9 ± 17.7	
4 +	461	62.3 ± 17.0	
**Socioeconomic position**			< 0.001
1 (wealthiest)	356	56.3 ± 14.5	
2	718	57.9 ± 15.8	
3	805	60.9 ± 16.3	
4 (poorest)	195	66.2 ± 19.1	
**Leisure-time maternal PA practice before pregnancy**			0.11
Yes	398	58.2 ± 15.2	
No	2205	59.6 ± 16.4	
**Leisure-time maternal PA during pregnancy**			0.02
Yes	104	55.9 ± 14.7	
No	2499	59.5 ± 16.3	
*Variables collected at 4 years of age*			
**Leisure-time maternal PA**			0.02
Yes	411	57.8 ± 15.3	
No	2113	59.8 ± 16.4	
**Maternal perception of child's PA**			< 0.001
Active	1608	60.3 ± 16.5	
Inactive	925	57.8 ± 15.5	
*Variables collected at 6 years of age*			
**Maternal schooling**			< 0.001
No schooling	26	66.9 ± 16.1	
Elementary school	1251	61.3 ± 16.7	
High school	943	58.0 ± 15.9	
University degree	343	55.5 ± 14.1	

PA – Physical activity.

**Table 4:** Multiple linear regression among overall physical activity at 6 years and predictors. 2004 Pelotas (Brazil) Birth Cohort Study.

Variables	β (mg)	95% CI	P
*Variables collected at birth*^a^			
**Sex**			< 0.001
Boys	(ref)	–	
Girls	− 8.65	(− 10.0; − 7.30)	
**Type of delivery**			0.35
Normal	(ref)	–	
Cesarean	− 0.67	(− 2.06; 0.72)	
**Low birthweight (< 2500** **g)**			0.35
Yes	(ref)	–	
No	− 1.18	(− 3.65; 1.30)	
**Preterm delivery**			0.83
No	− 0.25	(− 2.55; 2.04)	
Yes	(ref)	–	
**Parity**			0.14
1	(ref)	–	
2	0.51	(− 1.19; 2.20)	
3	− 0.02	(− 2.00; 1.97)	
4 +	2.20	(− 0.24; 4.16)	
**Socioeconomic position**			< 0.001
1 (wealthiest)	− 9.69	(− 12.45; − 6.93)	
2	− 8.10	(− 10.60; − 5.60)	
3	− 5.18	(− 7.62; − 2.64)	
4 (poorest)	(ref)	–	
**Maternal PA before pregnancy**			0.35
Yes	0.91	(− 1.01; 2.83)	
No	(ref)	–	
**Maternal PA during pregnancy**			0.33
Yes	− 1.81	(− 5.47; 1.84)	
No	(ref)	–	
*Variables collected at 4 years of age*			
**Maternal PA**			0.61
Yes	− 0.48	(− 2.32; 1.36)	
No	(ref)	–	
**Maternal perception of child's PA**			0.18
Active	1.75	(− 0.34; 3.17)	
Inactive	(ref)	–	
*Variables collected at 6 years of age*			
**Maternal schooling**^b^			< 0.001
No schooling	(ref)	–	
Elementary school	− 4.27	(− 10.36; 1.83)	
High school	− 7.14	(− 13.30; − 0.97)	
University degree	− 9.50	(− 15.82; − 3.17)	

a Includes additional adjustment for maternal age.

b No adjustment for socioeconomic position due to collinearity.
